# Cell line specific alterations in genes associated with dopamine metabolism and signaling in midbrain dopaminergic neurons derived from 22q11.2 deletion carriers with elevated dopamine synthesis capacity

**DOI:** 10.1016/j.schres.2022.05.010

**Published:** 2024-11

**Authors:** Matthew J. Reid, Maria Rogdaki, Lucia Dutan, Bjørn Hanger, Kaarin Sabad, Roland Nagy, Dwaipayan Adhya, Simon Baron-Cohen, Grainne McAlonan, Jack Price, Anthony C. Vernon, Oliver D. Howes, Deepak P. Srivastava

**Affiliations:** a Department of Basic and Clinical Neuroscience, The Maurice Wohl Clinical Neuroscience Institute, Institute of Psychiatry Psychology and Neuroscience, King's College London, London, UK; b MRC Centre for Neurodevelopmental Disorders, King's College London, London, UK; c Department of Child and Adolescent Psychiatry, Institute of Psychiatry Psychology and Neuroscience, King's College London, London, UK; d Department of Psychosis Studies, Institute of Psychiatry Psychology and Neuroscience, King's College London, London, UK; e Department of Forensic and Neurodevelopmental Sciences, Institute of Psychiatry, Psychology and Neuroscience, King's College, London, UK; f Autism Research Centre, Department of Psychiatry, University of Cambridge, Cambridge, UK

**Keywords:** Schizophrenia, Human induced pluripotent stem cells, Midbrain floor plate neural progenitor, [^18^F]-DOPA PET, Reprogramming

## Abstract

Microdeletions at the 22q11.2 locus are associated with increased risk for schizophrenia. Recent work has demonstrated that antipsychotic naïve 22q11.2 carriers display elevated levels of dopamine synthesis capacity (DSC) as assessed by ^18^F-DOPA PET imaging. While this is consistent with a role for abnormal dopamine function in schizophrenia, it is unclear what molecular changes may be associated with this neuro-imaging endophenotype, and moreover, if these alterations occur independently of clinical presentation. We therefore conducted a pilot study in which we generated human induced pluripotent stem cells (hiPSCs) from two 22q11.2 deletion carriers with elevated DSC *in vivo*, but distinct clinical presentations. From these and neurotypical control lines we were able to robustly generate midbrain dopaminergic neurons (mDA-neurons). We then assessed whether genes associated with dopamine synthesis, metabolism or signaling show altered expression between genotypes and further between the 22q11.2 deletion lines. Our data showed alterations in expression of genes associated with dopamine metabolism and signaling that differed between the two 22q11.2 hiPSC lines with distinct clinical presentations. This reinforces the importance of considering clinical, genetic and molecular information, when possible, when choosing which donors to generate hiPSCs from, to carry out mechanistic studies.

## 1 Introduction

The 22q11.2 deletion syndrome is caused by a hemizygous microdeletion in the 22q11.2 chromosome region and is associated with a highly variable clinical presentation (Morrow et al., 2018). Typical microdeletions are around 3 Mb in size, encompassing ~45 coding genes as well as 7 microRNAs (Morrow et al., 2018). Individuals carrying this microdeletion are at 40-fold greater risk of developing psychosis (Schneider et al., 2014) making 22q11.2 the strongest genetic risk factor for schizophrenia. Using ^18^F-DOPA PET, we recently showed that dopamine synthesis capacity (DSC) is significantly increased in antipsychotic naïve 22q11.2 deletion carriers (Rogdaki et al., 2021). Increased presynaptic striatal DSC has also been observed in individuals with schizophrenia (McCutcheon et al., 2018), predates the onset of psychosis and is directly associated with symptom severity (Egerton et al., 2013; Howes et al., 2013). Therefore, 22q11.2 deletion represents a genetically homogenous model holding potential for studying the molecular underpinnings of dopaminergic dysregulation in schizophrenia.

Despite the insights that animal models of 22q11.2 deletion have provided about dopaminergic function at molecular level (Chun et al., 2014; Zakharenko et al., 2015), human molecular phenotypes are less well understood. Dysregulation of genes associated with dopamine synthesis, storage and release has been identified in the midbrain of schizophrenic individuals in post-mortem studies, but not consistently reported (Howes et al., 2013; Perez-Costas et al., 2012; Purves-Tyson et al., 2017). Thus, the mechanism underlying dopamine dysregulation remains unclear. Human induced pluripotent cells (hiPSCs) retain the genetic background of the donor offering the unique advantage of allowing the study of potential molecular mechanisms underlying distinct and disease-relevant endophenotypes observed in subjects, in an *in vitro* system. In this pilot study, we aimed to bridge PET findings with *in vitro* disease models by generating midbrain dopaminergic neurons (mDA-neurons) from hiPSCs from two individuals carrying 22q11.2 deletions, both with high levels of DSC compared to control participants, but only one with a diagnosis of schizophrenia. Our study had two aims: a) to determine whether mDA-neurons from 22q11.2 carriers exhibit similar molecular changes as that seen in post-mortem studies, and b) whether mDA-neurons derived from 22q11.2 deletion carriers with distinct clinical presentations displayed similar or unique molecular changes in genes involved in dopamine synthesis, metabolism or signaling.

## 2 Materials and methods

Additional information on Material and Methods can be found in Supplemental Material.

### 2.1 Human induced pluripotent stem cells (hiPSCs) generation

Participants were recruited and methods carried out in accordance with the ‘Patient iPSCs for Neurodevelopmental Disorders (PiNDs) study’ (REC No 13/LO/1218). Informed consent was obtained from all subjects for participation in the PiNDs study. Ethical approval for the PiNDs study was provided by the NHS Research Ethics Committee at the South London and Maudsley (SLaM) NHS R&D Office. All hiPSC lines were generated from primary keratinocytes as described previously (Cocks et al., 2014). Keratinocytes were reprogrammed using the CytoTune-iPS 2.0 Sendai expressing Reprogramming Kit (ThermoFisher, A16517).

### 2.2 Generation of mDA-neurons

Generation of midbrain floor plate neural progenitor (mFPP) cells and dopamine (DA) neurons was based on (Fedele et al., 2017; Kriks et al., 2011) (Supplemental Fig. 2A). Briefly, hiPSCs were differentiated into mFFPs for 10 days before being expanded. Expanded mFFPs were then terminally differentiated into mDA-neurons until day 50. A detailed description of mDA-neuron generation can be found in Supplementary Material.

### 2.3 RT^2^ profiler PCR array

We used the Human Dopamine and Serotonin Pathway RT^2^ Profiler array (Qiagen) to assay the expression of 84 genes associated with dopamine and serotonin synthesis and signaling. RT-PCR was performed using the RT^2^ SYBR green qPCR Master Mix (Qiagen) using RNA from day 50 mDA-neurons using a BioMark HD cycler (Fluidigm). Analysis of gene expression was carried out using the RT^2^ profiler PCR array data analysis v3.5 software provided by Qiagen. All data was normalised to 5 separate housekeeping genes (*ACTB*, *B2M*, *GAPDH*, *HPRT1*, and *RPLP0*). The 2^ΔΔCT comparative method for relative quantification was used to quantify the genes expression. Three independent differentiations per hiPSC line were used in these experiments.

### 2.4 Statistical analysis

All statistical analysis was performed in GraphPad. Differences in 2^ΔΔCT, relative expression (Fold change) and cell number parameters were identified by comparisons between multiple conditions: main effects were probed by one-way-ANOVAs with Tukey or Bonferroni correction for multiple comparisons. Differences were considered significant if *P* was lower than 0.05 (*p* < 0.05). Error bars represent standard errors of the mean unless stated otherwise.

## 3 Results

### 3.1 Characterisation of 22q11.2 hiPSCs

HiPSCs were generated from two 22q11.2 deletion carriers: 22DM_287(+) and 22DF_191(−). Detailed clinical information can be found in Supplemental Information. Both 22q11.2 carriers had elevated DSC *in vivo*, as measured by ^18^F-DOPA PET (Supplemental Table 1 and 2) (Rogdaki et al., 2021). Participant 22DM_287(+) had a diagnosis of autism spectrum disorder, mild intellectual disability and schizophrenia. By contrast, participant 22DF_191(−) had cardiac defects but no diagnosis of psychiatric illness. For clarity, we use (+) following the cell line name to denote a diagnosis of schizophrenia, and (−) to indicate a lack of diagnosis. HiPSCs were confirmed as karyotypically normal; to express pluripotency markers and showed three germ layer differentiation (Supplemental Fig. 1A-D, Supplemental Fig. 2B, and Supplemental Table 1). For comparison we used HiPSCs from three (neurotypical) individuals with no history of psychiatric illness (CTR_M3; CTM_127; CTF_007) that have been previously characterized (Adhya et al., 2021).

**Fig. 1:**
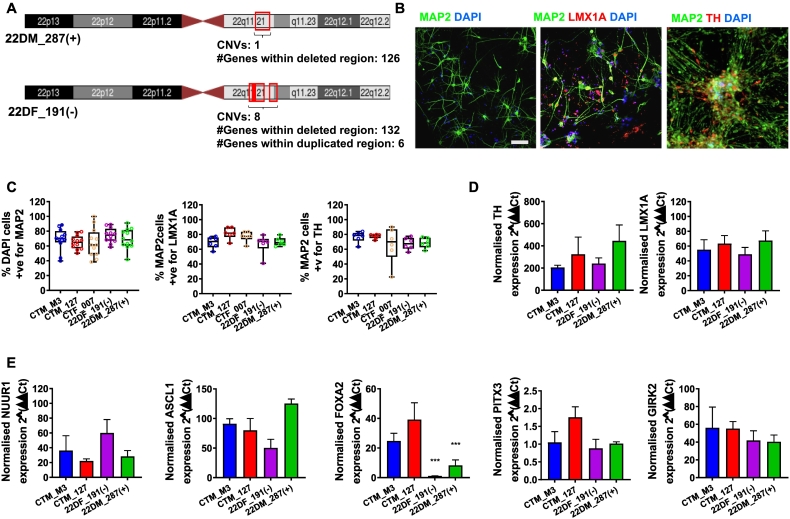
Generation of midbrain dopamine neurons from 22q11.2 deletion hiPSCs. (A) Schematic of the 22q11.2 CNVs and total number of coding gene affected in the hiPSC lines generated in this study. (B) Representative images of day 50 mDA-neurons generated from control and 22q11.2 deletion hiPSCs. After 50 days of differentiation mDA-neurons are positive for the pan-neuronal marker MAP2. In addition, mDA-neurons robustly express markers of dopamine identity, including LMX1A and tyrosine hydroxylase (TH). (C) Box and whisker blots represent quantification of DAPI cells positive MAP2; and MAP2 cells positive for LMX1A and TH in DA-neurons generated from control hiPSC lines (CTR_M3, CTM_127 and CTF_007) and 22q11 deletion hiPSC lines (22DF_191(−) and 22DM_287(+)). Error bars are represented as minimum and maximum values; each data point represents technical repeats from 3 independent experiments for each hiPSC line. (D & E) Quantitative PCR (qPCR) analysis of day 50 DA-neurons demonstrates expression of key genes inferring generation of midbrain dopaminergic neurons (*TH*, *LMX1A*, *NURR1*, *ASCL1*, *GRIK2*, *PITX3* and *FOXA2*). Comparisons between groups (hiPSC lines) was made using ANOVA with Bonfoerroni post-hoc analysis; *n* = 3 independent experiments per hiPSC line; error bars are represented as ±sem.

Single polymorphism (SNP) array (Infinium PsychArray) was used to determine genomic integrity, and to confirm the 22q11.2 deletion (Fig. 1A and Supplemental Fig. 3). The 22DM_287(+) line had a single hemizygous ~2.5 Mb deletion affecting 46 coding genes and 7 microRNAs (Supplemental Fig. 2). Cell line 22DF_191(−) had a total of eight copy number variants (CNVs) at the 22q11.2 locus. Of these, 4 were hemizygous deletions (~3.4 Mb in total); 1 was a homozygous deletion (~0.5 Mb), and 3 were duplications (~0.62 Mb in total). The deletions encompassed 41 coding genes and 6 microRNAs within the 22q11.2 locus, and 13 coding genes outside of this region. The duplicated regions included *ARVCF* and *TANGO2* genes, as well as the microRNA, *MIR185* (Supplemental Fig. 3). Genome-wide SNP genotype data were also used to derive schizophrenia polygenic risk score (PRS) using Psychiatric Genomics Consortium 3 genome wide association study (GWAS) summary statistics (Trubetskoy et al., 2022) for all hiPSC lines. This revealed that 22DM_287(+) had a higher adjusted PRS compared to 22DF_191(−) and all control lines, except for CTM_007, which also showed a high PRS (Supplemental Fig. 1E).

### 3.2 Generation of midbrain dopaminergic neurons from patient hiPSCs

After 50 days of differentiation, all hiPSCs, irrespective of genotype, generated mDA-neurons positive for MAP2 (>70%), LMX1A (>75%), a maker of midbrain dopamine ontogeny; and tyrosine hydroxylase (TH; >75%) (Fig. 1B-D). mDA-neurons from all genotypes also expressed mFPP specific transcription factors *NURR1*, *ASCl1* (Fig. 1E) as well as *GRIK2* and *PITX3* (Fig. 1E). Intriguingly, control mDA-neurons highly expressed *FOXA2*, whilst 22q11.2 mDA-neurons did not have detectable levels of *FOXA2* expression, irrespective of clinical presentation (Fig. 1E). Taken together, these data indicate that control and 22q11.2 hiPSC lines successfully differentiate into a midbrain dopaminergic neuronal fate.

### 3.3 mDA-neurons generated from 22q11.2 carriers with distinct clinical presentations, display differential expression of genes involved in dopamine synthesis and signaling

**Fig. 2:**
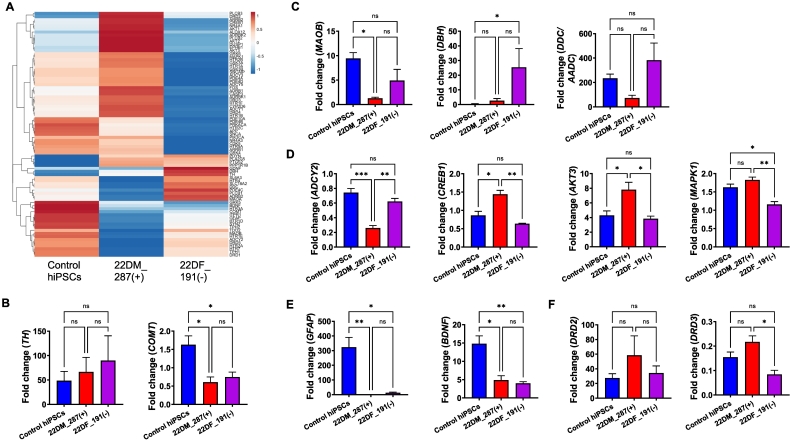
Expression of genes associated with dopamine synthesis, metabolism and signaling in mDA-neurons generated from control and 22q11.2 deletion hiPSCs. (A) Heatmap of expression values of genes assessed in PCR array clustered based on 2^ΔΔCT values. Data for all three control lines (CTR_M3, CTM_127 and CTF_007) were combined, whereas data for each 22q11.2 deletion line (22DF_191(−) and 22DM_287(+)) were assessed separately. Gene expression values were normalised to 5 housekeeping genes. (B) Normalised expression of *TH* and *COMT* in day 50 DA-neurons generated from control hiPSC lines and 22q11 deletion hiPSC lines. (C) Normalised expression of gene associated with dopamine synthesis and metabolism in day 50 DA-neurons generated from control hiPSC lines and 22q11 deletion hiPSC lines. (D) Normalised expression of gene associated with dopamine signaling in day 50 DA-neurons generated from control hiPSC lines and 22q11 deletion hiPSC lines. (E) Normalised expression of gene associated with dopamine targets in day 50 DA-neurons generated from control hiPSC lines and 22q11 deletion hiPSC lines. (F) Normalised expression of dopamine receptor expression in day 50 DA-neurons generated from control hiPSC lines and 22q11 deletion hiPSC lines. Comparisons between groups (hiPSC lines) was made using ANOVA with Bonfoerroni post-hoc analysis; *n* = 3 independent experiments per hiPSC line; error bars are represented as ±sem.

We next utilized a PCR array to profile the expression levels of genes involved in dopamine and serotonin synthesis and signaling from day 50 mDA-neurons. A total of 86 genes were assessed across all hiPSC lines. A summary of the main findings for each gene can be found in Table 1. We observed no difference in the expression of *TH* across all DA-neurons (Fig. 2A). As expected, 22q11.2 mDA-neurons expressed *COMT* at ~50% (Fig. 2A) given the hemizygosity for *COMT* gene in the 22q11.2 genomic region. These data indicated the reliability of the PCR array.

**Table 1:**
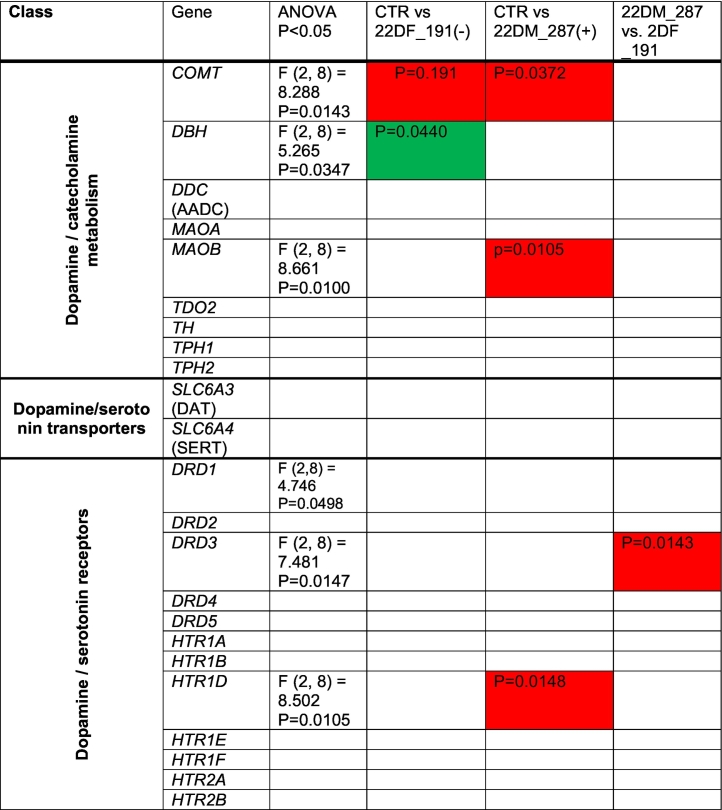

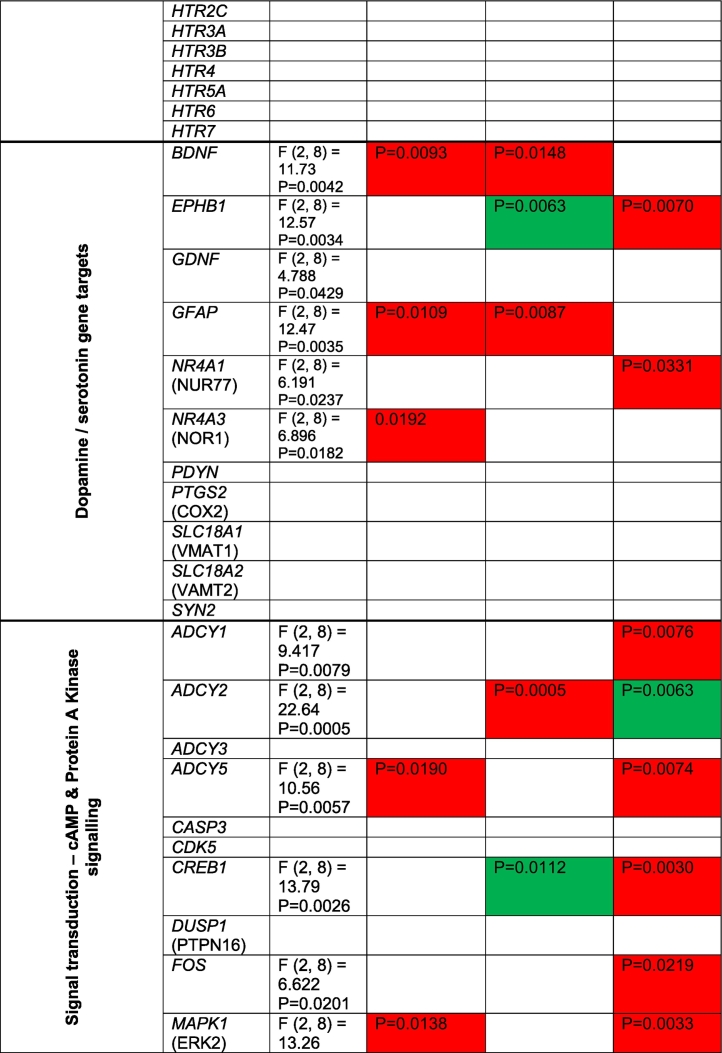

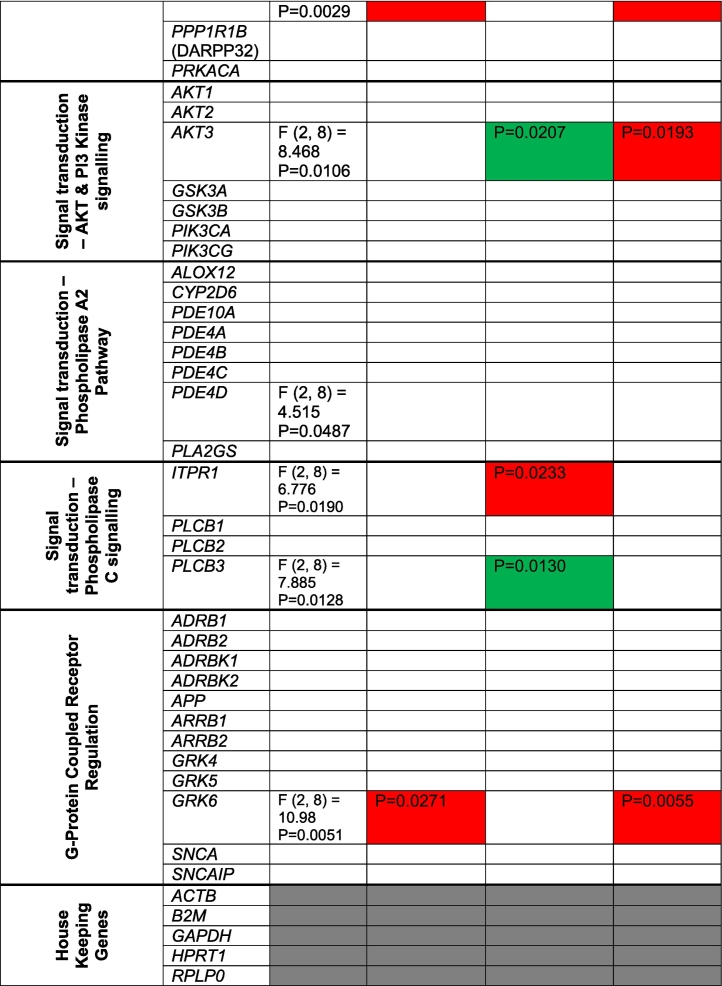
Summary of RT^2^ Dopamine and Serotonin PCR array Profiler. All 86 gene assessed (and housekeeper genes) are listed in day 50 DA-neurons from control (CTR) or 22q11.2 deletion (22DF_191(−); 22DM_287(+)) hiPSC lines. Columns named “CTR vs 22DF_191(-)” and “CTR vs 22DM_287(+)” are colour red or green to indicate expression of gene is either significantly upregulated or downregulated respectively, compared to control mDA-neurons.

Overall, 23 genes were differentially expressed in 22q11.2 mDA-neurons (Fig. 2A-F; Table 1). The majority of differentially expressed genes were those involved in dopamine synthesis/metabolism, receptor signaling and gene targets (Table 1) consistent with previous post-mortem studies (Purves-Tyson et al., 2017). Sixteen of these genes showed differential expression in only one 22q11.2 hiPSC line, which was of potential interest in light of the different clinical presentations. For example, mDA-neurons from 22DM_287(+) displayed reduced expression of *MAOB*, whereas mDA-neurons from 22DF_191(−) had elevated expression of *DBH* (Fig. 2B). Both genes are involved in conversion of dopamine into either DOPAC or noradrenaline; no difference in the expression of *MAOA* was observed (Fig. 2C). The expression of *DDC* (*AADC*) was not significantly different from control hiPSCs but trended towards elevated expression in 22DF_191(−) and lower expression in 22DM_287(+) mDA-neurons (Fig. 2C).

Of the genes involved in DA signaling, *ADCY2*, *CREB1* and *AKT3* were of note as these genes were only differentially expressed in the 22DM_287(+) mDA-neurons (Fig. 2D). All of these genes have been associated with increased risk for SCZ. Conversely, *MAPK1* was reduced expression in 22DF_191(−) DA-neurons, consistent with its location within a deleted region of the 22q11 locus in this cell line (Supplemental Fig. 2).

## 4 Discussion

In this pilot study, we aimed to identify molecular changes present in the mDA-neurons which may be relevant for this imaging endophenotype. We observed cell line specific dysregulation of genes involved in dopamine metabolism and signaling in mDA-neurons, with a greater number of genes showing differential expression in the 22q11.2 line generated from the individual with a diagnosis of schizophrenia compared to those from individuals with no psychiatric illness and a similar microdeletion.

The finding that *TH* expression is unaltered is consistent with previous studies in *COMT* deficient mice (Gogos et al., 1998; Huotari et al., 2002), and a post-mortem study of 22q11.2 deletion carriers (Butcher et al., 2013). Similarly, the expression of *COMT* at ~50% is consistent with this gene being within deletion CNVs within the 22q11.2 region of 22DM_287(+) and 22DF_191(−). Four genes involved in DA metabolism and signaling (*MAOB*, *ADCY2*, *CREB1*, *AKT3*) showed altered expression in 22DM_287(+) mDA-neurons only. Furthermore, expression levels of *DCC* (*AADC*) and *DBH*, two dopaminergic catabolic enzymes, displayed differential expression between 22DM_287(+) and 22DF_191(−). The observed decrease in expression of dopamine catabolic enzymes is in agreement with a previous report indicating a reduction of peripheral HVA in 22q11.2 deletion carriers (Boot et al., 2008). Thus, one possible explanation for these findings is that disruption of genes involved in dopamine metabolism and signaling may segregate between 22q11.2 deletion carriers with distinct clinical presentations. It is interesting to note that mDA-neurons generated from 22DF_191(-) had elevated expression levels of *DBH*, an enzyme that could degrade dopamine, and thus speculatively we suggest, may reflect a compensatory mechanisms in this hiPSC line to offset the increase in DA due to *COMT* heterozygosity. Another possibility is that differences between 22q11.2 lines may be due to additional genetic variants within the genome. Consistent with this, 22DM_287(+) hiPSCs had a higher PRS compared to 22DF_191(−) and all control hiPSC lines, with the exception of CTF_007, which has a similar PRS for schizophrenia to 22DM_287(+). It also important to note that 22DF_191(−) has 4 duplication CNVs in addition to deletions within the 22q11.2 region. Notably, duplications within the 22q11.2 region has been associated with lower risk for psychosis compared to the general population, suggesting a possible protective role (Rees et al., 2014). Therefore, it is possible that both molecular and clinical differences may be influenced by the presence of duplications at the 22q11.2 locus. Another area of importance that requires further investigation is the influence of biological sex in these findings. For example, sex-specific transcriptional differences have been observed in hiPSCs from schizophrenic individuals (Tiihonen et al., 2019), which may influence pathophysiology. In addition, *MAOB* and *MAOA* are located on the X chromosome, and thus may be subject to x-inactivation in females. Whether this is the case or not would impact the interpretation of gene expression data in the context of comparison between male and female 22q11.2 deletion carriers. Moreover, it will be important to consider whether sex also drives the increased DSC levels seen in 22DF_191(-): recent work indicates that women showing greater DSC capacity than men (Nordio et al., 2022).

Previous hiPSC studies using neurons derived from 22q11.2 deletion carriers have demonstrated robust alterations in microRNAs expression, mitochondrial deficits and altered functional properties in forebrain-like neurons (Khan et al., 2020; Li et al., 2021; Zhao et al., 2015), as well as PERK dysfunction in mDA-neurons (Arioka et al., 2021). Clinical phenotypes associated with 22q11.2 deletion, however, are highly variable and not all of the aforementioned studies have used hiPSCs from 22q11.2 deletion subjects with the same clinical presentation. Cellular phenotypes associated with 22q11.2 deletion show variable penetrance, segregating with clinical presentation (Li et al., 2021). It is, therefore, important to consider if any observed cellular and molecular phenotypes relate to the 22q11.2 deletion and/or the associated clinical phenotype (Khan et al., 2020) or other variants within the genome. We have explored this further by generating hiPSCs from 22q11.2 deletion individuals, who have distinct clinical presentations, but a common neuro-imaging endophenotype in elevated DSC. We have subsequently looked for common or distinct molecular phenotypes associated with dopamine synthesis or signaling. Of note, our study used two 22q11.2 deletion hiPSCs lines, and therefore future studies using larger number of lines are required to confirm our findings. Nevertheless, our preliminary results reinforce the importance of considering clinical as well as genetic and molecular information, where possible, when choosing which donors to generate hiPSCs for mechanistic studies relevant understanding genotype-phenotype associations in 22q11.2 deletion carriers.

## Role of funding

This paper represents independent research part funded by the National Institute for Health Research (NIHR) Mental Health Biomedical Research Centre (BRC) at South London and Maudsley NHS Foundation Trust and King's College London. The views expressed are those of the author(s) and not necessarily those of the NHS, the NIHR or the Department of Health and Social Care.

## Contributions

Conceptualization, D.P.S., A.C.V., M.R & O.D.H.; Methodology, D.P.S., A.C.V. M.J.R. & L.D.; Experimental Investigation and Data Analysis, M.J.R., L.D. R.N., K.S., B.H., D.A. & D.P.S.; Contribution of Unique Resources, J.P., M.R, S.B-C. & G.McA.; Writing, M.R, D.P.S., & A.C.V.; Supervision, D.P.S.

## Declaration of competing interest

The authors declare no competing interests.

## Appendix A Supplementary data

Supplementary material Image 1
