# Supplementary material for: Cell line specific alterations in genes associated with dopamine metabolism and signaling in midbrain dopaminergic neurons derived from 22q11.2 deletion carriers with elevated dopamine synthesis capacity
Source: Schizophr Res. 2024 Nov;273:98–106. doi: 10.1016/j.schres.2022.05.010 (PMC11586776; doi:10.1016/j.schres.2022.05.010)
Supplement: Supplementary file 1 — Supplementary material [file mmc1.pdf]

Matthew J. Reid[1,2$], Maria Rogdaki[1,3,4$], Lucia Dutan[1,2], Bjørn Hanger[1,2], Kaarin Sabad[1,2], Roland Nagy[1,2], Dwaipayan Adhya[1,6], Simon Baron-Cohen[6], Grainne McAlonan[2,5], Jack Price[1,2], Anthony C. Vernon[1,2#], Oliver D. Howes[2,4#], Deepak P. Srivastava[1,2#*].

[$] = equal contribution

[#] = joint senior authors

* = corresponding author: deepak.srivastava@kcl.ac.uk

Supplemental Material:

Supplemental Materials and Methods

Supplemental Results

Supplemental Tables

Supplemental Materials and Methods

Clinical information on the individuals with 22q11.2 deletion

Participants were recruited and methods carried out in accordance with the 'Patient iPSCs for Neurodevelopmental Disorders (PiNDs) study' (REC No 13/LO/1218). Informed consent was obtained from all subjects for participation in the PiNDs study. Ethical approval for the PiNDs study was provided by the NHS Research Ethics Committee at the South London and Maudsley (SLaM) NHS R&D Office.

Participant 22DM_287(+) was a male individual (30 years old) carrying a 22q11.2 deletion. This individual had a diagnosis of autism spectrum disorder and mild intellectual disability. During the clinical assessment, he scored highly in prodromal psychotic and anxiety symptoms, as measured with the Comprehensive Assessment of at-Risk Mental state and Beck's Anxiety scale respectively (Beck et al., 1988; Yung et al., 2005). In addition, this individual displayed elevated levels of dopamine synthesis capacity as determined by [18F]-DOPA PET, compared to healthy controls (Rogdaki et al., 2021). Six months following the PET scan, this individual developed psychosis and later on received the diagnosis of schizophrenia. He was not on any medication at the time of the scanning.

Participant 22DF_191(-) was a female individual (19 years old) carrying a 22q11.2 deletion, and who had cardiac defects. She had unremarkable past psychiatric history and did not present with any significant psychopathology during the assessment. This individual also displayed elevated levels of dopamine synthesis capacity as determined by [18F]-DOPA PET, compared to healthy controls (Rogdaki et al., 2021). For further details, please **Supplementary Table 1**.

Reagents

The following antibodies were used: anti-MAP2 chicken polyclonal (Abcam; AB104896); anti-LMX1A rabbit polyclonal (Abcam; AB139726); anti-tyrosine hydroxylase (TH) rabbit polyclonal (Millipore; AB152); anti-COMT rabbit monoclonal (Abcam; AB126618); anti-SMA rabbit polyclonal (Abcam; AB5694); anti-AFP mouse monoclonal (Millipore; AB3980); anti-Nanog rabbit polyclonal (Abcam; AB80892); anti-OCT4 rabbit polyclonal (Life Technologies; 701756); anti-SSEA4 mouse monoclonal (Life Technologies; MA0121); anti-TRA-1-81 mouse monoclonal (Life Technologies; MA0124); anti-Tuj1 (TUBB3) mouse monoclonal (BioLegend; 801201).

Generation of human induced pluripotent stem cells (hiPSCs)

All hiPSC lines were generated from primary keratinocytes as described previously (Cocks et al., 2014). Briefly, 1 x 10^5 primary hair root keratinocytes were reprogramed by introducing OCT4, SOX2, KLF4 and C-MYC factors with a CytoTune-iPS 2.0 Sendai expressing Reprogramming Kit (ThermoFisher, A16517). Transformed keratinocytes were plated onto an irradiated MEF feeder layer (Millipore) supplemented Epilife medium for ten days before switching to 'hES media', which consisted of KO-DMEM/F12 supplemented with 20% Knock-out serum replacement, Non-essential amino acids, Glutamax, β-mercaptoethanol (all from Life Technologies) and bFGF (10 ng/ml; Peprotech) (KOSR media). After a further two weeks, reprogrammed colonies were selected and plated on Geltrex (Life technologies) coated Nunc treated multidishes (Thermo Scientific) into E8 media (Life Technologies). hiPSCs reprograming was validated by genome-wide expression profiling using Illumina Beadchip v4 and the bioinformatics tool 'Pluritest' **(Supplemental Figure 1A)**. Additionally, the tri-lineage differentiation potential was stablished by embryoid body formation; immunocytochemistry (ICC) to validate the

expression of different pluripotency markers including Nanog, OCT4, SSEA4 and TRA1-81 and the alkaline phosphatase activity by Alkaline phosphatase expression kit (Milipore) **(Supplemental Figure 1B-D)**. Genome integrity was assessed by an Illumina Human CytoSNP-12v2.1 beadchip array (~300,000 markers) and analyzed using KaryoStudio software (Illumina). hiPSCs were incubated in hypoxic conditions at 37°C and maintained in E8 media replaced every 24 hours until the cells monolayer reach ~95% confluence. A summary of hiPSC lines used in this study can be found in **Supplementary Table 2**.

Neuronal differentiation

Generation of midbrain floor plate neural progenitor (mFPP) cells from hiPSC lines and subsequent generation of dopamine (DA) neurons was performed as outlined in (Fedele et al., 2017; Kriks et al., 2011) **(Supplemental Figure 2A)**. Briefly, neuronal differentiation of 30% confluent iPSCs was initiated by replacing Essential 8 medium (Gibco) with 'neuralization media' composed of KnockOut Serum Replacement (KOSR) medium (Gibco) supplemented with various small molecules to induce neuralization (Fedele et al., 2017; Kriks et al., 2011).). Cells were maintained at 37°C in normoxic conditions in neuralization medium for 10 days. During this period, media was replaced every 24 hours whilst transitioning from base KOSR into N2 medium (Gibco) and altering small molecules. Media was supplemented with 10 μM SB431542 (Sigma-Aldrich) and 250 ng/ml LDN193189 (Sigma-Aldrich) on day 1. Subsequent media on days 2-4 contained additional 2 μM purmorphamine (Sigma-Aldrich), 50 μg/ml smoothened agonist (SAG; Sigma-Aldrich) 100 ng/ml Fibroblast growth factor 8 (FGF8) and 3 μM CHIR99021 (Sigma-Aldrich). SB43 was removed from day 5. SAG and FGF8 were removed from day 8. Purmorphamine was removed from day 9. On

day 10, mFPPs were passaged using Accutase (Life Technologies) and re-plated in N2 media supplemented with 250 ng/ml LDN193189 (Sigma-Aldrich) and 3 μM CHIR99021 (Sigma-Aldrich) only (mFPP expansion-media). Cells were passaged and expanded a further three times at reduced ratios. Cells were cryopreserved in mFPP expansion-media with 10% DMSO for future use. For terminal plating of mFFPs into dopamine neurons (DA-neurons), cells were passaged or thawed and plated as a single cell suspension at low density onto 0.6 μg/cm^2 poly-D-lysine (Gibco), 1 μg/cm^2 fibronectin (Sigma-Aldrich) and 2 μg/cm^2 laminin (Sigma-Aldrich) coated Nunc Cell-Culture treated multidishes (Thermo Scientific) in B27 media (Gibco) supplemented with 20 ng/ML BDNF (Peprotech), 20 ng/ml GDNF (Perpotech), 200 μM AA2P (Sigma-Aldrich), 0.5 mM cAMP (Sigma-Aldrich), 1 ng/ml TGFβ (Miltenyi Biotec) and 10 μM DAPT (Santa Cruz Biotechnology). Media was then changed every other day until day 50 when they were used for experimentation. During all passaging and terminal plating steps 10 μM Rock Inhibitor Y-27632 (Sigma Aldrich) was added to aid cell survival.

qRT-PCR

Total RNA harvested and lysed with Trizol reagent (Life technologies) and isolated by centrifugation with 100% Chloroform, following by 100% isopropanol and lastly by 75% ethanol. The RNA was purified by precipitation with 100% ethanol and Sodium acetate (Life technologies) and quantify with the NanoDrop 1000 Spectrophotometer (Thermo scientific). Residual genomic DNA was removed by addition of TURBO DNA-free (Life technologies) and incubation at 37°C for 30 minutes. Complementary DNA (cDNA) was synthesized from 1 μg of total RNA from each extraction using random hexamer primers and SuperScript III (Life Technologies) following the manufacturer's recommendations. qPCR was performed with HOT FIREPol EvaGreen qPCR Mix Plus

ROX (Solis Biodyne) carried out according to the manufacturer's instructions in a total volume of 20 µl, containing 1:5 diluted cDNA, qPCR mix and primers at to a final concentration of 0.3 µM. PCR reaction conditions: 95°C for 15 minutes for the initial denaturation followed by 95°C for 30 seconds, 60°C for 30 seconds and 72°C for 30 seconds during 33 cycles. The melting curve analyses was preformed from 60°C to 95°C with readings every 1°C. The 2^ ΔΔCT comparative method for relative quantification was used to quantify the genes expression. Three independent differentiations per hiPSC line were used in these experiments. The data CT values were normalized to GAPDH, RPL27 and SDHA housekeeping genes. Primer sequences can be found in **Supplementary Table 3**.

RT^2 Profiler PCR array

To assess the expression profile of dopamine-related genes in DA-neurons derived from hiPSC lines, we used the Human Dopamine and Serotonin Pathway RT^2 Profiler array (Qiagen) on the BioMark HD (Fluidigm). This assay profiles the expression of 84 genes associated with dopamine and serotonin systems. Generation of cDNA and subsequent preamplification of RNA isolated from day 50 DA-hiPSC-neurons, was performed using the RT^2 PreAMP cDNA Synthesis Kit and RT^2 PreAMP Pathway Primer Mixes kits (Qiagen). Following preamplification, samples were diluted with nuclease-free water and added to the RT^2 SYBR green qPCR Master Mix (Qiagen); loaded onto reaction plates and the real-time amplification data (Ct values) were determined using a BioMark HD cycler (Fluidigm). Analysis of gene expression from real-time results was carried out using the RT^2 profiler PCR array data analysis v3.5 software provided by Qiagen. Each array contained 5 separate housekeeping genes (*ACTB*, *B2M*, *GAPDH*, *HPRT1*, and *RPLP0*) that were used for normalization of the

sample data. Normalization to the house keeping genes (HKG) was performed by calculating the ΔCt for each gene of interest (GOI) in the plate (Ct value of GOI-Ct value of HKG). Any Ct value >35 was considered to be a negative call. The 2^ ΔΔCT comparative method for relative quantification was used to quantify the genes expression. Three independent differentiations per hiPSC line were used in these experiments. The differential expression analyses were performed by using a one-way ANOVA with Bonferroni correction and 95% confidence interval with the Prism package of GraphPad software. The heatmap was generated by using the log2-transformed 2^ ΔΔCT values using hierarchical clustering methods with the packages pheatmap and rcolourbrewer in RStudio.

Immunocytochemistry (ICC)

Treated DA-neurons were fixed with 4% formaldehyde plus 4% sucrose in PBS. Fixed neurons were permeabilized in 0.1% Triton-X-100 in PBS for 15 minutes and blocked in 4% normal goat serum in PBS for 1 hour at room temperature. Primary antibodies were added to the block solution in an antibody dependent concentration and incubated overnight at 4°C. Immunoreactivity was achieved by incubating the cells with 1:500 concentration of Alexa Fluor 594 conjugated anti-mouse IgG, Alexa Fluor 594 conjugated anti-goat IgG and Alexa Fluor 488 conjugated anti-rabbit IgG in block buffer. For nuclei staining a 1:2000 concentration of DAPI (Thermo Fisher) was used.

Imaging of immunofluorescence by high content image screening

mFPPs were plated at a density of $1x10^4$ cells/well on poly-D-lysine and laminin-coated optical-bottom 96 well plates with polymer base (ThermoScientific). mFPPs were differentiated into DA-neurons, grown until day 50, fixed and processed for ICC as

described above. Image acquisition was performed using an Opera Phenix High Content screening platform (Perkin Elmer): images were acquired using a 20 x (NA 0.4) objective. The Harmony High Content Imaging and Analysis Software was used to determine the number of DAPI or MAP2 cells positive for specific markers. For each hiPSC line, 2 biological replicates with 3 technical replicates per condition were imaged and analyzed: 15 randomly selected fields from each technical replicate was examined. Data from each technical replicate was used as a single data point and compared between each biological replicate and each hiPSC line. The means of percentages of positive cells were compared by an ANOVA.

Statistical analysis

All statistical analysis was performed in GraphPad. Differences in $2^{\Delta\Delta CT}$, relative expression (Fold change) and cell number parameters were identified by comparisons between multiple conditions: the main effects and simple effects were probed by one-way-ANOVAs with Tukey or Bonferroni correction for multiple comparisons. Differences were considered significant if *P* was lower than 0.05 ($p < 0.05$). Error bars represent standard errors of the mean unless stated otherwise.

Supplemental Results

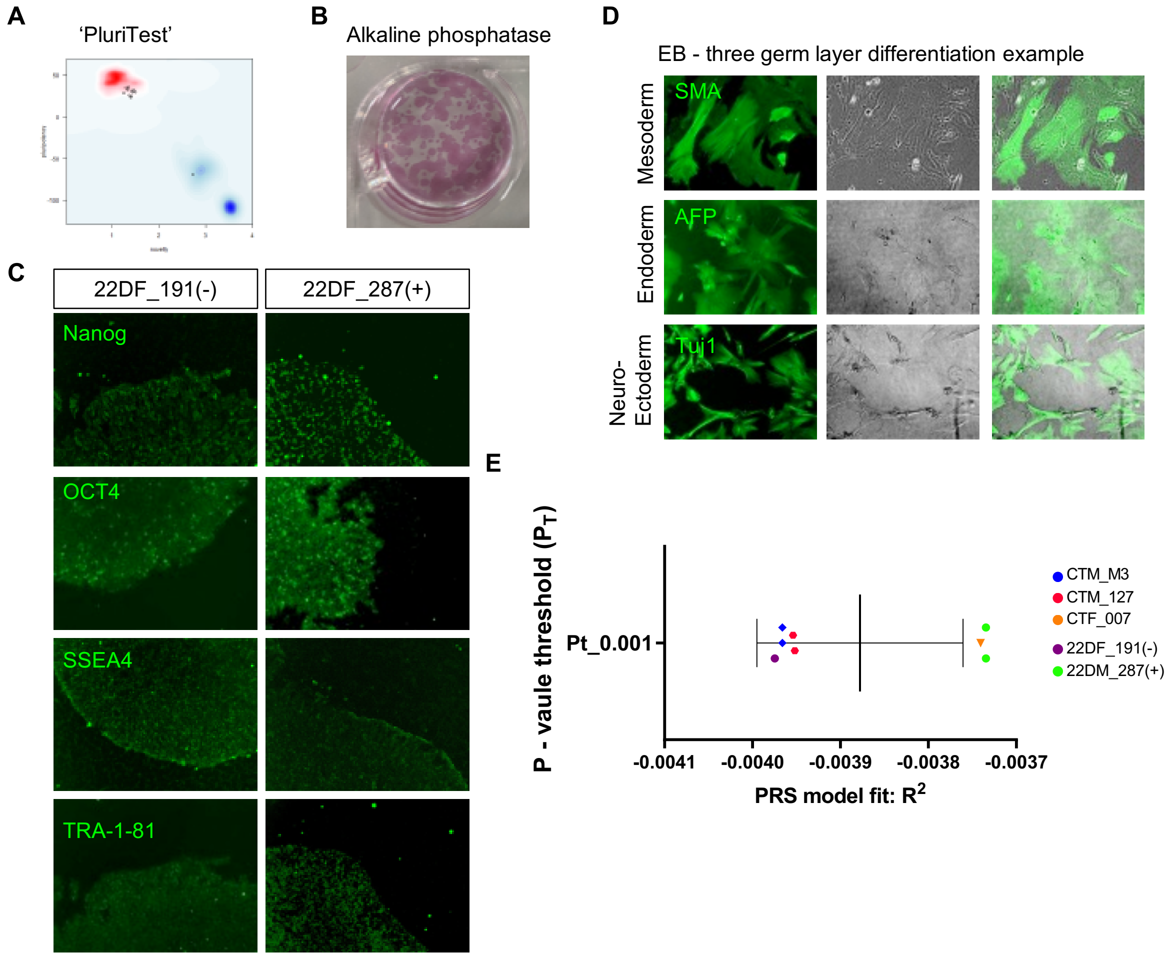

Supplemental Figure 1. Characterisation of hiPSC lines generated from patient hair keratinocytes. (A) PluriTest analysis of Illumina HT12v4 transcriptome array data was used to determine that hiPSC generated cluster with pluripotent stem cells (red cloud) and not with partly- or differentiated cells (blue clouds). **(B)** Representative example of positive Alkaline phosphatase activity of hiPSCs. **(C)** Example of ICC validation of pluripotency of hiPSC lines using antibodies against Nanog, OCT4, SSEA4 and TRA1-81. **(D)** Representative example of tri-lineage differentiation potential by embryoid body formation. **(E)** Predicted polygenic risk score for schizophrenia based on Psychiatric Genomics Consortium (PGC) 3 GWAS summary

statistics, using PRSice (Euesden et al., 2015) and genome-wide SNP genotype data of control and 22q11.2 deletion lines.

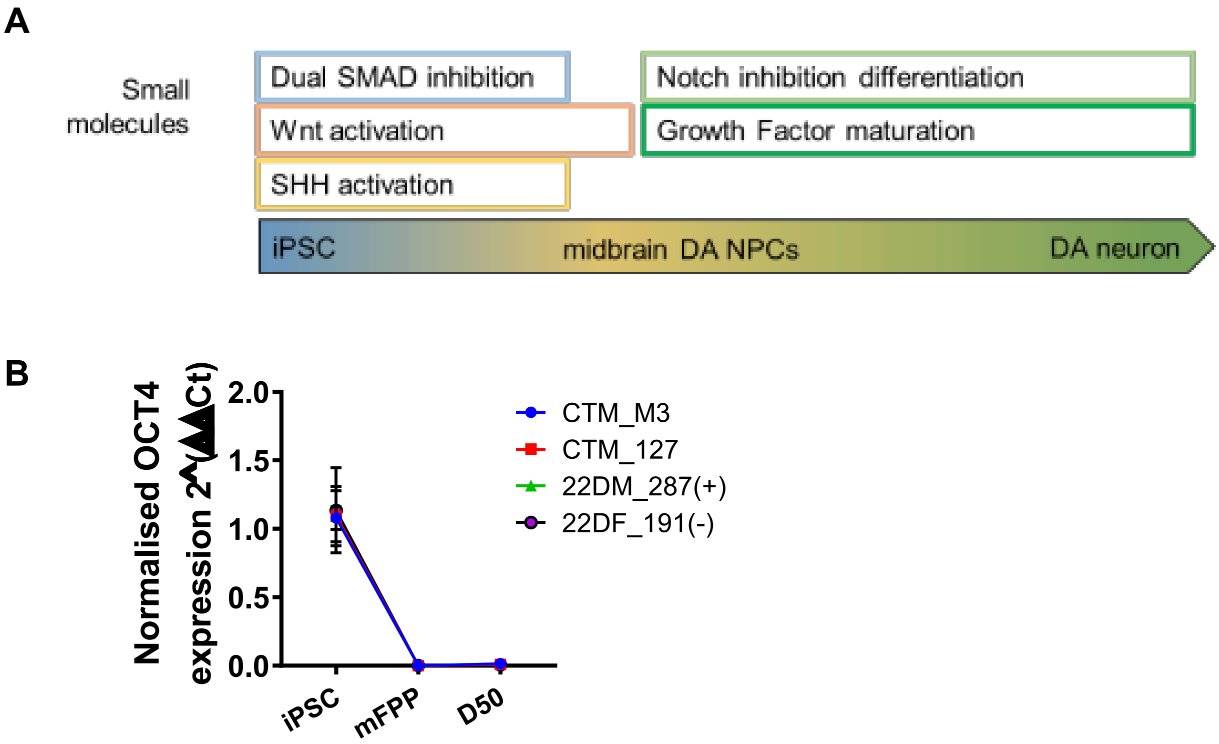

Supplemental Figure 2. Generation of mDA-neurons from control and 22q11.2 deletion hiPSC lines. (A) Schematic overview of differentiation protocol used in study**. (B)** QPCR assessment of *OCT4* expression levels in undifferentiated hiPSCs, mFPP and day 50 mDA-neurons.

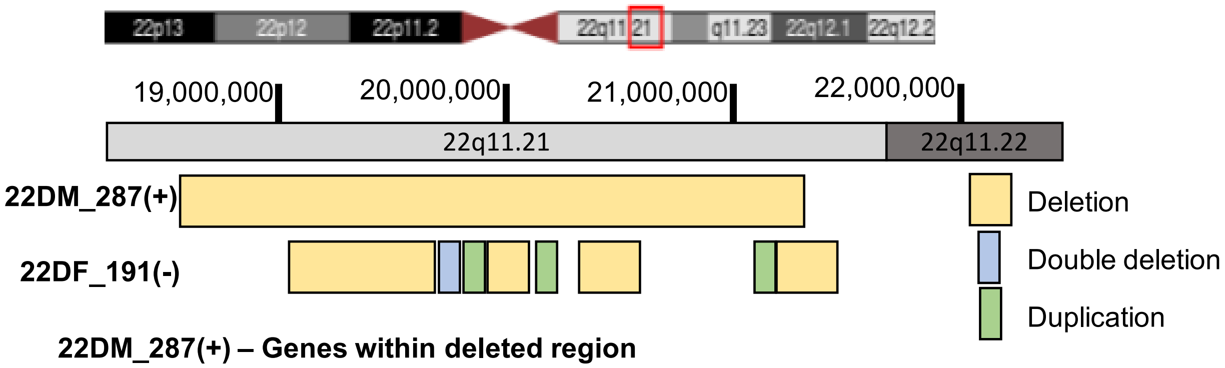

22DM_287(+) – Genes within deleted region

FAM230F	CLTCL1	TBX1	MIR1306	USP41	AIFM3
DGCR6	HIRA	GNB1L	TRMT2A	ZNF74 -	LZTR1
PRODH	MRPL40	TXNRD2	MIR6818	SCARF2	THAP7
DGCR5	C22orf39	COMT	RANBP1	KLHL22	P2RX6
DGCR9	UFD1	MIR4761	ZDHHC8	LOC1019288 24	SLC7A4
DGCR2	CDC45	ARVCF	CCDC188	MED15	MIR649
DGCR11	CLDN5	TANGO2	LINC00896	PI4KA	POM121L7P
TSSK2	LINC00895	MIR185	RTN4R	SERPIND1	RIMBP3B
LINC01311	SEPTIN5	DGCR8	MIR1286	SNAP29	HIC2
SLC25A1	GP1BB	MIR3618	DGCR6L	CRKL	

46 coding genes
7 microRNA
5 lncRNAs

22DF_191(-) – Genes within deleted regions

TSSK2	CLDN5	DGCR8	USP41	SNAP29	TMEM191C	PPM1F
GSC2	LINC00895	MIR3618	ZNF74 -	CRKL	RIMBP3C	
LINC01311	CCDC188	MIR1306	SCARF2	AIFM3	UBE2L3	TOP3B
SLC25A1	SEPTIN5	TRMT2A	KLHL22	P2RX6	YDJC	
CLTCL1	GP1BB	MIR6818	LZTR1	MIR649	CCDC116	
HIRA	RTN4R	RANBP1	THAP7	LRRC74B	SDF2L1	
MRPL40	TBX1	ZDHHC8	MED15	POM121L7P	MIR301B	
C22orf39	GNB1L	LINC00896	SLC7A4	YPEL1	MIR130B	
UFD1	COMT	MIR1286	PI4KA	RIMBP3B	PPIL2	
CDC45	MIR4761	DGCR6L	SERPIND1	HIC2	MAPK1	

41 coding genes
6 microRNAs
2 lncRNAs
13 Coding genes outside of classic deletion

22DF_191(-) – Genes within duplicated regions

ARVCF	MIR185
TANGO2	

2 coding genes
1 microRNA

Supplemental Figure 3. Characterisation of CNVs in 22q11.2 locus in 22q11.2 deletion hiPSC lines. Using genome-wide SNP genotype data from 22q11.2 deletion hiPSC lines, CNVs within the 22q11.2 chromosomal regions were calculated.

Supplementary Table 1. Ki^{cer} estimates for the whole striatum and for the striatal subdivisions for a) the carriers of 22q11.2 deletion from whom hiPSCs were derived, b) 22q11.2 deletion carriers, c) healthy control group, as provided by Rogdaki et al, 2021. Ki^{cer} estimates for groups b and c are provided in mean (SD).

**Ki^{cer} estimates**				
	**22DM_287(+)**	**22DF_191(-)**	**22q11.2 deletion group**	**Healthy controls**
**Whole striatum**	0.01599	0.01468	0.0143 (0.0012)	0.0127 (0.001)
Functional subdivisions				
**associative**	0.01628	0.01476	0.0145 (0.0013)	0.0128 (0.0011)
**sensorimotor**	0.015	0.0145	0.0139 (0.001)	0.0125 (0.001)
**limbic**	0.0168	0.0147	0.0146 (0.0013)	0.013 (0.0011)

Supplemental Table 2. Summary of hiPSC lines used in study.

iPSC line	Clone	Age range	Diagnosis	Sex	CytoSNP-12v2.1	EB differentiation	'PluriTest'
**CTF_007**	10	30-50	Apparently Healthy	Female	Pass	Yes	Pass
**CTM_M3**	36S	30-50	Apparently Healthy	Male	Pass	Yes	Pass
**CTM_127**	03	30-50	Apparently Healthy	Male	Pass	Yes	Pass
**22DF_191(-)**	13	15-30	22q11.2 DS - none	Female	Pass	Yes	Pass
**22DM_287(+)**	01	30-50	22q11.2 DS - autism spectrum disorder; mild intellectual disability; schizophrenia	Male	Pass	Yes	Pass

Supplemental Table 3. Summary of qPCR primers used in study.

Primer name	Primer sequence (5' to 3')
**FOXA2 F**	GAGCCCGAGGGCTACTCC
**FOXA2 R**	GCCCACGTACGACGACAT
**LMX1A F**	AGAGCTCGCCTACCAGGTC
**LMX1A R**	AGAAGGAGGCCGAGGTGT
**POU5F1 F**	ATCCAGTCCCAGGACATCAA
**POU5F1 R**	TGGTCGTTTGGCTGAATACC
**ASCL1 F**	GGACGAGGGCTCTTACGAC
**ASCL1 R**	GATCACCCTGCTTCCAAAGT
**GIRK2 F**	GGAACTGGAAATTGTGGTCAT
**GIRK2 R**	CATCACCATTTCTTTCTGTTT
**TH F**	GCGGTTCATTGGGCGCAGG
**TH R**	CAAACACCTTCACAGCTCG
**NESTIN F**	ACCAAGAGACATTCAGACTCC
**NESTIN R**	CCTCATCCTCATTTTCCACTCC
**PITX3 F**	GGACTAGGCCCTACACACAGA
**PITX3 R**	TCCGCGCACGTTTATTTC
**NURR1 F**	CGACATTTCTGCCTTCTCC
**NURR1 R**	GGTAAAGTGTCCAGGAAAAG

References

Beck, A.T., Epstein, N., Brown, G., Steer, R.A., 1988. An inventory for measuring clinical anxiety: psychometric properties. Journal of consulting and clinical psychology 56(6), 893.

Cocks, G., Curran, S., Gami, P., Uwanogho, D., Jeffries, A.R., Kathuria, A., Lucchesi, W., Wood, V., Dixon, R., Ogilvie, C., Steckler, T., Price, J., 2014. The utility of patient specific induced pluripotent stem cells for the modelling of Autistic Spectrum Disorders. Psychopharmacology (Berl) 231(6), 1079-1088.

Euesden, J., Lewis, C.M., O'Reilly, P.F., 2015. PRSice: Polygenic Risk Score software. Bioinformatics 31(9), 1466-1468.

Fedele, S., Collo, G., Behr, K., Bischofberger, J., Muller, S., Kunath, T., Christensen, K., Gundner, A.L., Graf, M., Jagasia, R., Taylor, V., 2017. Expansion of human midbrain floor plate progenitors from induced pluripotent stem cells increases dopaminergic neuron differentiation potential. Sci Rep 7(1), 6036.

Kriks, S., Shim, J.W., Piao, J., Ganat, Y.M., Wakeman, D.R., Xie, Z., Carrillo-Reid, L., Auyeung, G., Antonacci, C., Buch, A., Yang, L., Beal, M.F., Surmeier, D.J., Kordower, J.H., Tabar, V., Studer, L., 2011. Dopamine neurons derived from human ES cells efficiently engraft in animal models of Parkinson's disease. Nature 480(7378), 547-551.

Rogdaki, M., Devroye, C., Ciampoli, M., Veronese, M., Ashok, A.H., McCutcheon, R.A., Jauhar, S., Bonoldi, I., Gudbrandsen, M., Daly, E., van Amelsvoort, T., Van Den Bree, M., Owen, M.J., Turkheimer, F., Papaleo, F., Howes, O.D., 2021. Striatal dopaminergic alterations in individuals with copy number variants at the 22q11.2 genetic locus and their implications for psychosis risk: a [18F]-DOPA PET study. Molecular Psychiatry.

Yung, A.R., Yuen, H.P., McGorry, P.D., Phillips, L.J., Kelly, D., Dell'Olio, M., Francey, S.M., Cosgrave, E.M., Killackey, E., Stanford, C., Godfrey, K., Buckby, J., 2005. Mapping the onset of psychosis: the Comprehensive Assessment of At-Risk Mental States. The Australian and New Zealand journal of psychiatry 39(11-12), 964-971.
